# The Effect of a Fully Immersive Virtual Reality Training Program on Walking Parameters in People With Unilateral Below‐Knee Amputation: An Experimental and a Single‐Subject Study

**DOI:** 10.1002/hsr2.71974

**Published:** 2026-05-17

**Authors:** Reza Vahab Kashani, Mokhtar Arazpour, Akbar Biglarian, Fatemeh Keshavarzi

**Affiliations:** ^1^ Iranian Research Center on Aging University of Social Welfare and Rehabilitation Sciences Tehran Iran; ^2^ Orthotics and Prosthetics Department University of Social Welfare and Rehabilitation Sciences Tehran Iran; ^3^ Department of Biostatistics and Epidemiology, Social Departments of Health Research Institute University of Social Welfare and Rehabilitation Sciences Tehran Iran; ^4^ Student Research Committee University of Social Welfare and Rehabilitation Sciences Tehran Iran

**Keywords:** amputation, gait training, lower limb amputee, rehabilitation, virtual reality, walking speed

## Abstract

**Background and Aims:**

The virtual reality (VR) environment has the potential to be an effective component of walking training in the rehabilitation of lower limb amputees. Creating an accessible walking training program based on fully immersive VR environment could enhance the applicability of this technology in this population.

**Methods:**

This is a single‐subject ABA design study consisted of two parts. The first part focused on developing a VR video and evaluating its immersion and user experience among ten individuals with transtibial amputations. The second part was an ABA‐style single‐subject study involving two participants with a new traumatic transtibial amputation. These participants used the developed VR environment for 6 weeks. Outcome measures were including the 10‐m walk test, spatiotemporal gait parameters, and presence, before the VR training and after the 6 weeks training. All measurements were repeated every other day during four experimental sessions in baseline, immediately after intervention and 4 weeks after end of intervention to ensure the stability of reported variables frequency.

**Results:**

The quality of the fully immersive VR video was evaluated by ten amputee participants. The mean scores for presence, entertainment, and immersion exceeded half of the maximum score, while the comfort score did not reach half. After 6 weeks of intervention, the 10‐m walk test showed speed improvement by a mean increase of 0.3 m/s. The duration of the stance and loading response phases increased in both limbs. Step and stride lengths exhibited an increase corresponding to the enhanced velocity of walking. The immersion score exhibited a one and half point increase before and after the 6 weeks of intervention.

**Conclusion:**

A fully immersive virtual reality video‐based walking training program is well‐accepted by transtibial prosthetic users and demonstrates potential to impact gait parameters and improve walking asymmetry in individuals with transtibial amputation.

**Abbreviations**
d‐index: The effect size function automatically displays the percent change and calculated values for both the ES and d‐index for any two phases. Information for interpreting calculated values appears in the Console.
g‐index: The g‐index is a measure of effect size calculated using the proportion of scores in the desired zone. Used when there is a trend in the data. The g‐index is a measure of effect size calculated using the proportion of scores in the desired zone. Used when there is a trend in the data.

## 1 Introduction

Walking is our most essential and natural way to get around in our everyday lives. It's not just a means of transportation; it's a rhythmic dance of movement that connects us to our surroundings and keeps us active throughout the day. Walking is a multifactorial function and relates to physical and mental health.

Lower limb amputation significantly impacts the gait cycle and is a leading cause of disability worldwide [1]. It highlights the critical need for gait rehabilitation in this population. Enhancing walking performance is among the primary goals of rehabilitation for lower limb amputees [2].

Walking is affected by multiple factors, including balance, vision, and muscle control and muscle function. Enhanced motor control mechanisms in lower limb amputees [3] can significantly improve the effectiveness of walking rehabilitation programs. Motor control refers to the management of movements in organisms with a nervous system. The motor control exhibits a complex interplay of movements that encompasses both voluntary and involuntary responses. These movements include conscious actions, the automatic execution of learned motor skills, and reflex actions that occur without conscious thought. This intricate system highlights the remarkable capabilities of our nervous system and muscular function.

Engaging in mental exercises can enhance motor control. Research indicates that “mental training” of movements may result in muscle activation, likely through action potentials and contractions. The results of studies on mental exercises in people with walking disorders have been promising [4]. Based on previous studies, limb amputation in humans does not prevent a person's mental imagery, but it disrupts the clarity of this mental imagery [5]. However, Mental movements decrease after lower limb amputation, especially leg movements on the amputated side. When imagining movements, the body's autonomic system also activates the same energy sources needed to perform real movements [6]. For example, the heart rate and respiratory frequency during actual walking and mental training at different speeds increase in the same way as the walking speed increases. Mental exercises are as important as physical exercise.

The more relevant and realistic the input provided to the brain during exercise, the more valuable the training becomes, and the greater the likelihood that this integrated sensory information will help to optimally reorganize the brain [7]. Visual information can strongly signal the reorganization of sensorimotor circuits, and the deliberate observation of activities can effectively engage motor and premotor areas of the brain. The human brain can undergo significant physiological, functional, and structural changes over time, which may facilitate the restoration of lost functions after injury and amputation [8]. New technologies, such as virtual reality, can enhance neuroplasticity by providing a suitable environment [9]. Acquiring and practicing new skills is crucial for promoting neuroplastic changes and improving recovery after neurological damage [7].

Virtual reality (VR) is a technology that simulates a computer‐generated environment, allowing for realistic interaction with it. It has become increasingly popular in recent years, with applications in gaming, education, and healthcare. Rehabilitation based on virtual reality improves motor learning. Examining the effectiveness of virtual reality‐based rehabilitation for simulating movement training of an artificial hand in healthy adults showed that motor learning is enhanced through movement imitation [10].

Immersion is a fundamental aspect of an engaging interactive video [11]. In VR, immersion describes how effectively an application, experience, or technology captivates users by creating a rich sensory and interactive environment. While it is related to the concept of presence in VR, the two are not quite the same. Fully immersive virtual reality aims to transport users into a computer‐generated world, giving them the sensation that they have truly stepped inside this synthetic environment. Currently, one of the most lifelike settings is achieved through 3D camera videos, which can be displayed using VR head‐mounted displays (HMDs) or through multiple projection systems.

Fully immersive VR mind–body exercises contribute to increasing mental health and physical function. Moreover, integrating VR mind–body exercises with various forms of physical activity can create a dynamic approach to enhancing overall health [12]. The use of virtual environments is one approach that helps lower limb amputees train in a simulated environment. The fully immersive VR training had positive effects on basic cognitive skills associated with motor tasks practiced during the intervention games [13].

The fully immersive visual training method was still not applied to gait training for individuals with lower‐limb amputations. Research indicates that mental movements are a dynamic process [14]. While lower‐limb amputations are more prevalent than those of the upper limbs [1], most studies in the area of mental movement primarily focus on upper‐limb amputations.

The fully immersive VR potential to create a virtual reality environment for gait training can significantly enhance rehabilitation for individuals with unilateral lower limb amputations. However, developing software in an artificial environment that works in conjunction with a robot to assist with walking, as previous studies have shown, will increase costs and reduce the chances of incorporating this approach into regular rehabilitation practices in clinics.

So, the aim of this study was to develop a fully immersive VR‐based training program to improve lower limb amputees' walking rehabilitation and evaluate the effects of this program on spatiotemporal gait parameters and walking performance in transtibial amputees after 4 weeks of intervention.

## 2 Material and Methods

### 2.1 Ethics statement

This research protocol was approved by the Ethics Committee of the University of Social Welfare and Rehabilitation Sciences. We received the approval code (IR.USWR.REC.1402.107) On December 21, 2023. Participant recruitment began on March 6, 2024, and continued until August 2024 at the University of Social Welfare and Rehabilitation Sciences based on the guidelines of the Declaration of Helsinki. All participants provided written informed consent before their involvement in the study.

#### 2.1.1 Participants

A previous sample size estimation was performed utilizing G*Power 3.1. In order to evaluate the primary outcome, 12 participants were required to attain 80% power (α = 0.05, effect size = 0.40) [15].

Twelve persons with recent unilateral below‐knee amputation participated in this study via a call to Tehran city hospitals. This single‐subject study randomly selected two participants from a pool of 12 based on pre‐defined inclusion and exclusion criteria [16]. The two participants were included in the training program evaluation. The demographic data of the included participants are reported in Table 1. The inclusion criteria were being a man or woman in the age range of 20–59 years old with a unilateral traumatic transtibial amputation that happened during the past 2 weeks.

**Table 1:** Participants demographic data.

Case number	Age	Height (cm)	Weight (kg)	Body mass index	Residual limb length	Gender	Amputated side	Comorbidity	Foot type
1	50	170	70	24.2	24	Man	Left	—	SACH
2	22	178	82	25.9	21	Man	Right	—	DESR
3	36	170	76	26.3	15	Woman	Right	—	SACH
4	53	175	66	21.6	18	Man	Left	—	DESR
5	50	168	69	24.4	14	Man	Right	—	DESR
6	52	180	95	29.3	22	Woman	Right	—	SACH
7	54	187	106	30.3	20	Man	Right	Diabetes	DESR
8	38	162	61	23.2	16	Woman	Right	—	DESR
9	52	170	73	25.3	18	Woman	Left	Diabetes	SACH
10	43	190	104	28.8	25	Man	Left	—	DESR
11*	48	176	73	23.6	19	Man	Right	—	DESR
12*	50	173	71	23.7	11	Man	Right	—	DESR
Mean ± SD	45.24 ± 9.36	175.42 ± 8.04	79.42 ± 14.69	25.6 ± 2.66	19.17 ± 3.41				

* Participant included to the second phase of the study.

#### 2.1.2 Inclusion Criteria

Participants were eligible if they were first‐time users of trans‐tibial lower limb prosthetics.

Participants were eligible if they faced with traumatic amputation.

Individuals were excluded if they encountered:

‐ complete blindness or other visual impairments,
‐ other associated conditions such as cancer, Parkinson's disease, neurological diseases (e.g., stroke, traumatic brain injury, Alzheimer's),
‐ any condition that interferes with a motor exercise program (e.g., intermittent claudication)
‐ any joint disease, including arthritis or lower limb joint contractures,
‐ any symptoms of motion sickness
‐ Also, participants were excluded if they received less than a 24/30 score on the mental state test.

#### 2.1.3 Preparing the Stump to Receive the Prosthesis

Approximately 8 weeks after amputation and healing of sutures, participants received a transtibial prosthesis. The socket design and suspension system ensured participants' comfort and acceptance during the study [17]. Then, all participants intended in a 2‐week stump care and rehabilitation program of physiotherapy and occupational therapy five times a week to reach the optimal level of their functional capacities [18]. Each physiotherapy session lasts for 90 min and includes learning how to wear the prosthesis and check the condition of the stump, carrying out weight transfer exercises and training motor capacities and proprioception. Occupational therapy sessions lasted for 1 h and mainly included teaching how to perform activities of daily living, such as climbing stairs, getting up from a lying position, moving weight, being in public, and transporting or driving a car. To limit the effect of prosthetic foot type on walking parameters [19], we used dynamic energy storing and return (DESR) foot with the same type in all participants, which does not significantly affect gait parameters during normal walking [20, 21].

#### 2.1.4 VR Video Development

The virtual immersive 360° video is captured with a VR camera (Insta 360 × 2 VR camera). The video camera mounted on the forehead of a healthy person recorded their walking in two different environments at normal human walking speeds (between 1.2 and 1.4 m/s) [22]. The first part of the video was recorded on a walking path inside the park. The second part was captured in a grassy field. Throughout the video, participants could hear the sounds of children playing in the park and birds chirping in the field. Both videos were captured on a Flat ground without slope during the day. Video recordings were conducted under 1920 × 1080 rectilinear resolution and a frame rate of 60 fps to ensure smooth playback in VR glasses. Data collected from the camera were processed using the Adobe Premiere Pro software (24.6.1.2 version) to accommodate VR glasses. The 4 K VR video was uploaded to a virtual reality glass (Meta Quest 2, 1832 × 1920 resolution per eye, refresh rate 120 Hz) and synced with the researcher's tablet computer. It helped the researcher to have the user's vision during the intervention. The VR video was validated for compatibility with the Meta Quest glasses, confirming 4 K resolution and proper encoding through playback testing and comparisons with existing videos featuring defined characteristics.

#### 2.1.5 Intervention

**Figure 1:**
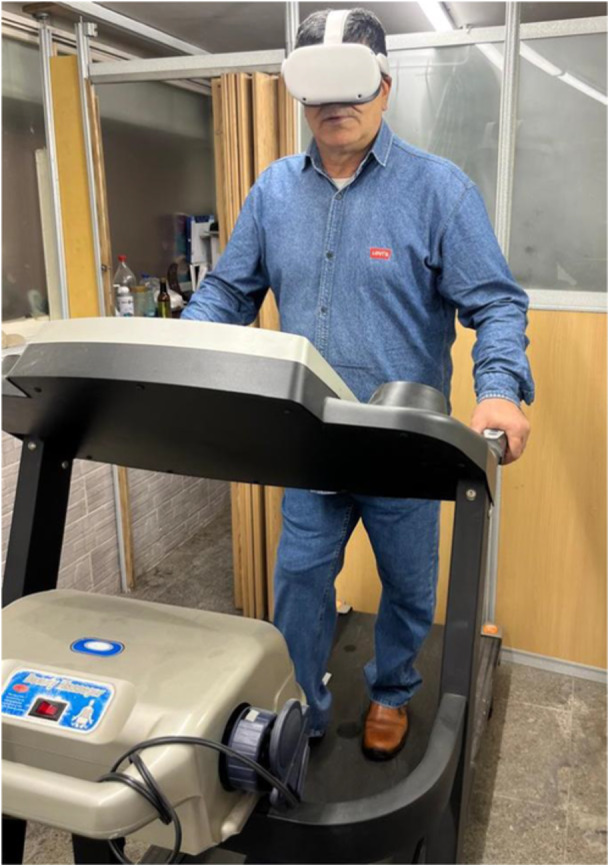
Participants walking on a treadmill while wearing a VR headset.

After participating in a 2‐week stump care and rehabilitation program, 10 participants were included in the initial part of the study to evaluate the feasibility of VR video training. All participants walked for 1 min on a treadmill with normal speed. Then, the treadmill speed was chosen based on each participant's self‐selected normal walking speed. Participants walked on the treadmill at self‐selected normal speed while wearing the VR glasses for 5 min (Figure 1). The researcher stood close to the treadmill for any assistance needed while taking care of participants and focused on the vision and speed of walking. Participants could increase their walking speed if they want during the training. After 5 min of training with a treadmill and VR glasses, participants had some rest and started to answer questions about their VR training experience.

Two participants of a single‐subject study after a pre‐intervention assessment (4 weeks before intervention = phase A) were trained with a developed VR video and treadmill for 6 weeks, three times per week, for 1 h each time, with a sequence of 5 min of walking in parallel and 5 min of rest that was the phase B. Then both participants were evaluated during the 4 weeks of follow up (phase A1).

#### 2.1.6 Outcome Measures

The VR training experiences were evaluated using a checklist designed specifically for this study. The questions focused on user comfort and performance indicators related to the VR training program. User comfort indicators were developed based on existing research on the comfort of electronic wearables [23]. These included factors such as comfort while wearing and using the glasses, ease of understanding the training program, the weight of the glasses, and overall acceptance of the technology. Ten participants assessed these comfort indicators using a visual continuous scale.

The research employed a tailored questionnaire informed by previous studies evaluating presence in virtual reality. This questionnaire encompassed aspects such as comfort, entertainment, presence in virtual reality, and immersion, aimed at capturing the participants' experiences using the virtual reality glasses [24]. The questions primarily featured a mix of comfort ratings, a group attendance questionnaire, and an individual attendance questionnaire. Additionally, participants completed the I‐GROUP test, which assessed their understanding of the virtual environment and their ability to visualize themselves within VR environment [25].

Each participant responded to the questions using a VAS after the examiner posed them. The examiner clarified the questions as needed to ensure understanding. The questionnaire comprised 16 questions covering areas like immersion, comfort, entertainment, and presence, with responses recorded for each participant. These were measured on a four‐point VAS, resulting in a total score across all sections. Detailed descriptions of the verbal questions were reported in the appendix.

The outcomes of this single‐subject study focused on gait parameters. The examiner evaluated the walking speed with the 10‐meter walking test. This test is a reliable and valid assessment tool used in various conditions, including lower limb amputation. Participants walked on a straight path along a 10‐m sidewalk at maximum walking speed. We ignored the first and last two meters of the walking. It excludes the acceleration and deceleration time [26]. The walking speed was defined by dividing the distance by the walking time measured with a stopwatch.

The gait parameters were evaluated via the Noraxon Myo motion system. This system is a valid and reliable wearable device for evaluating walking parameters in various conditions [27], including amputation. Seven wireless inertial sensors with 3‐axis accelerometers were positioned on the shoes, shanks, thighs and waist [28]. Spatio‐temporal parameters recorded in each test included speed (m/s), stride length (cm), and cadence (steps/min).

The visual experience was assessed with a visual analog scale (VAS) during the 6‐week intervention. Right after a 5‐min session, participants rated their immersion level to gauge the immersive quality of the video. They rated the immersive experience on a scale from 0 to 4 by responding to four specific questions in the initial phase of the study.

We repeated the assessment of all outcome measures 4 weeks before the VR training, during the intervention and 4 weeks after training, continuing this process until we observed stability in the records.

#### 2.1.7 Statistical Analysis

The normality of the participants' demographic data was assessed with the Shapiro‐Wilk test. The results section provides descriptive statistics for the initial phase of the study. Data from the ABA single‐subject design study were analyzed using the SSDforR package [29]. RStudio, with R version 4.4.3, was utilized for this analysis. Researchers evaluated the participants' data through descriptive tests. Subsequently, they examined trends and autocorrelation, applying moving averages and first differencing methods for data improvement when necessary. Effect sizes were calculated and reported using two methods of effect size calculation in SSDforR package. d‐index or g‐index depending on the observed trends. Finally, ANOVA and Tukey's test were conducted to determine the significance of the intervention effects [29].

## 3 Results

All participants lost their dominant lower limb. Participants' detail of demographic data includes age (45.24 ± 9.36), height (175.42 ± 8.04), weight (79.42 ± 14.69), body mass index (25.6 ± 2.66) and residual limb length (19.17 ± 3.41) were reported in Table 1.

The immersion questions were categorized into four parts, including Immersion, comfort, entertainment and the presence. The mean scores for presence (12.6 ± 1.43), entertainment (12.5 ± 2.07), immersion (13.5 ± 2.12), and comfort (7.2 ± 0.92) were reported in Table 2.

**Table 2:** Details of scores on VR‐based walking training experience for each participant.

Case number	Presence	Entertainment	Immersion	Comfort
1	11	10	10	7
2	14	12	12	7
3	13	11	14	6
4	11	12	16	8
5	12	11	11	6
6	12	12	14	8
7	15	15	13	8
8	14	11	13	8
9	13	16	16	8
10	11	15	16	6
Mean ± SD	12.6 ± 1.43	12.5 ± 2.07	13.5 ± 2.12	7.2 ± 0.92

**Figure 2:**
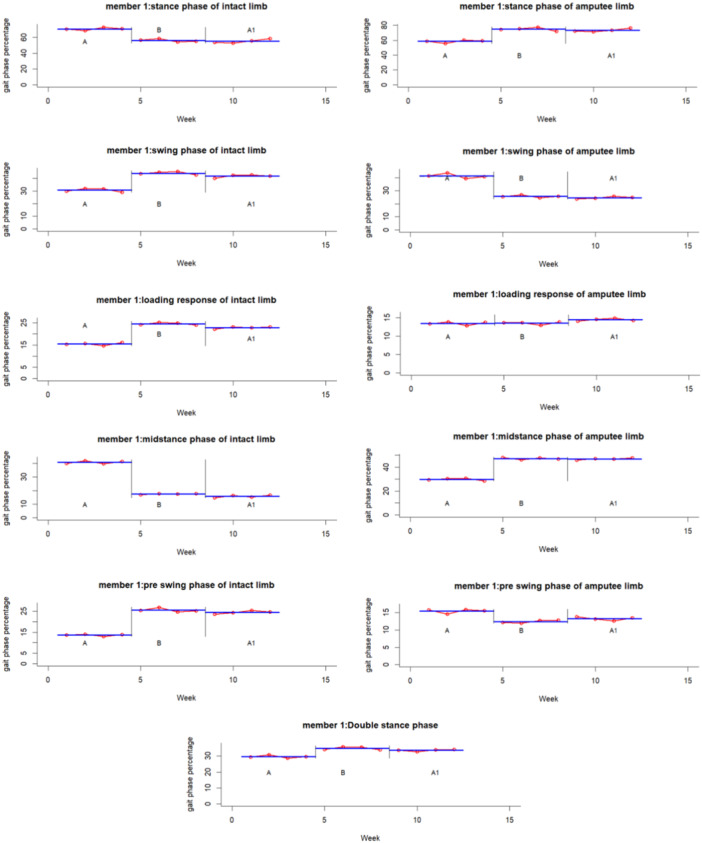
Gait phase parameters percentage of participant number one including: stance phase, swing phase, loading response, midstance, pre‐swing, and double stance phase.

**Figure 3:**
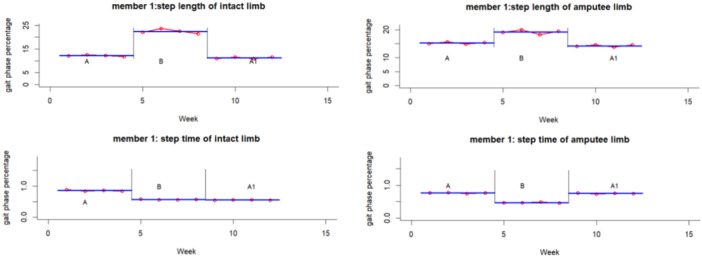
Gait phase parameters percentage of participant number two, including: stance phase, swing phase, loading response, midstance, pre‐swing and double stance phase.

**Figure 4:**
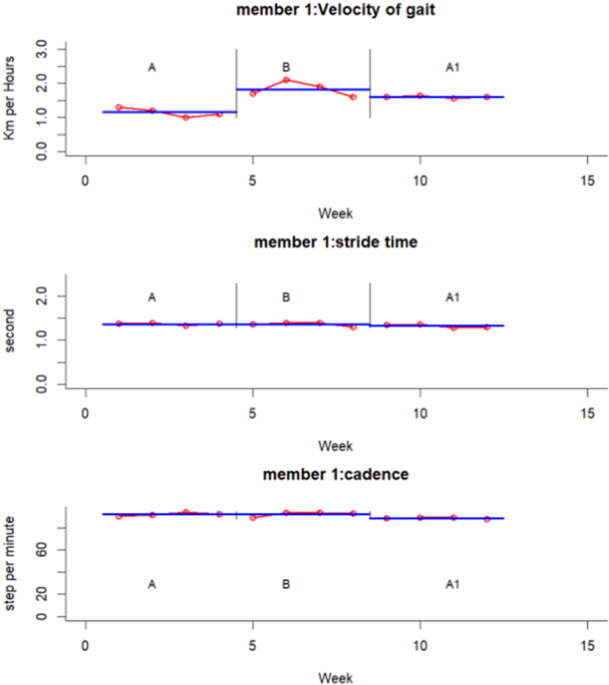
Spatiotemporal gait parameters of participant number one including step length and step time.

**Figure 5:**
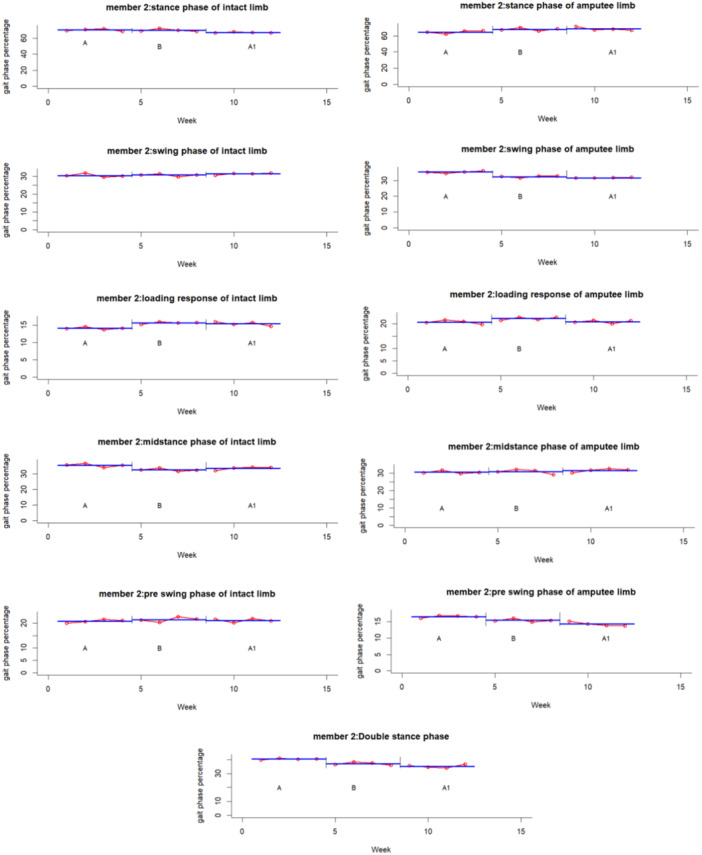
Spatiotemporal gait parameters of participant number two, including step length and step time.

**Figure 6:**
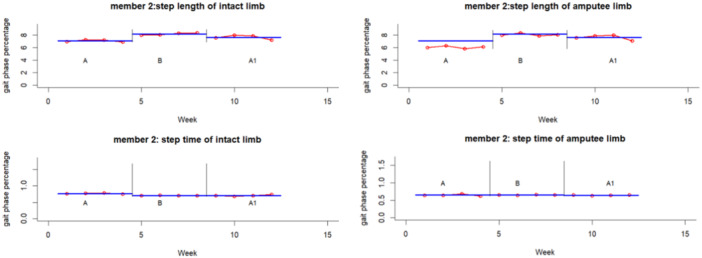
Spatiotemporal gait parameters of participant number one including velocity, stride time, and cadence.

**Figure 7:**
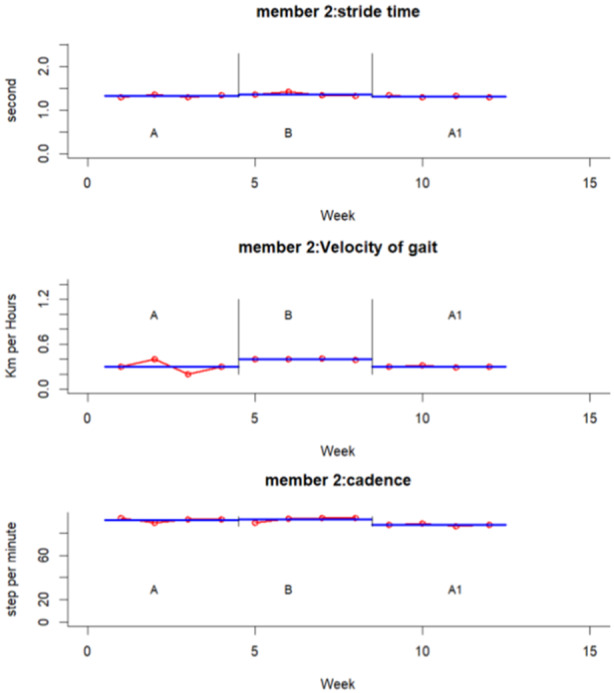
Spatiotemporal gait parameters of participant number two including velocity, stride time and cadence.

The experimental results for each participant and variable are summarized graphically in Figures 2, 3, 4, 5, 6, 7, which visualize temporal and spatial gait parameter fluctuations. Figures 2 and 3 present normalized data on gait cycle phase durations, expressed as percentages of the total cycle. Figures 4 and 5 quantify temporal variations in step time and spatial changes in step length. Figures 6 and 7 detail dynamic adaptations in locomotor performance, including velocity, cadence, and stride time measurements. All graphical representations adhere to standardized biomechanical reporting conventions for human motion analysis.

Data collected during the pre‐ and intra‐intervention swing phases for the amputated limb, as well as step length measurements of the intact limb in Participant 2, showed non‐stationary trends. To address these trends, we applied data transformations using a moving average filter, followed by first‐order differencing to stabilize the variance. Autocorrelation function (ACF) analyses were conducted, confirming stationarity, with no significant autocorrelation detected in any phase (all *p* > 0.05). Descriptive statistics for the raw gait parameters are detailed in Table 3, while standardized effect sizes are presented in Table 4. The d‐index was used to quantify differences between the intervention and baseline means, normalized by the pooled standard deviation. Since three gait phases required detrending prior to analysis, effect sizes were calculated solely from the detrended datasets. A d‐index threshold of > 2.67, which indicates large effects, was used to interpret clinical relevance, aligning with conservative benchmarks in rehabilitation biomechanics.

**Table 3:** Descriptive data of evaluations.

		B	A1	B	A1	B	A1	CV
variable	Case and limb	Mean	Mean	SD	SD	Range	Range	
Stance phase %	Case1‐intact limb	56.12	55.10	1.88	2.45	(54.3–58.6)	(52.9–58.4)	0.034
	Case1‐amput limb	74.92	73.52	2.22	2.04	(72.1–77.5)	(71.6–76.3)	0.03
	Case2‐intact limb	70.05	67.20	1.67	0.74	(68.4–72.3)	(66.5–68.2)	0.024
	Case2‐amput limb	67.95	68.57	1.78	2.04	(65.8–70.0)	(66.9–71.5)	0.026
%Load response	Case1‐intact limb	24.50	22.72	0.53	0.49	(24.0–25.1)	(22.0–23.1)	0.022
	Case1‐amput limb	13.52	14.47	0.43	0.35	(12.9–13.9)	(14.1–14.9)	0.032
	Case2‐intact limb	15.70	15.45	0.29	0.58	(15.3–16.0)	14.7–16.0)	0.019
	Case2‐amput limb	22.02	20.72	0.62	0.62	(21.3–22.6)	(19.9–21.3)	0.029
Mid stance %	Case1‐intact limb	17.55	15.87	0.37	0.93	(17.0–17.8)	(14.8–16.8)	0.022
	Case1‐amput limb	47.25	46.92	0.90	0.90	(46.2–48.1)	(45.8–48.0)	0.019
	Case2‐intact limb	32.70	33.65	0.94	1.07	(31.6–33.9)	(32.1–34.5)	0.029
	Case2‐amput limb	30.85	31.55	1.30	1.04	(29.1–32.1)	(30.1–32.6)	0.042
Pre swing %	Case1‐intact limb	25.52	24.50	0.97	0.73	(24.6–26.9)	(23.6–25.4)	0.038
	Case1‐amput limb	12.47	13.27	0.44	0.51	(12.0–12.9)	(12.6–13.8)	0.036
	Case2‐intact limb	21.40	21.07	0.96	0.75	(20.3–22.6)	(20.1–21.8)	0.045
	Case2‐amput limb	15.42	14.27	0.49	0.59	(14.9–16.1)	(13.8–15.1)	0.032
Swing phase %	Case1‐intact limb	44.07	41.75	1.25	1.15	(42.6–45.3)	(40.1–42.6)	0.028
	Case1‐amput limb	25.65	24.57	0.96	0.84	(24.6–26.9)	(23.6–25.6)	0.038
	Case2‐intact limb	30.70	31.42	0.69	0.57	(29.8–31.5)	(30.6–31.9)	0.023
	Case2‐amput limb	32.47	31.78	0.65	0.20	(31.5–32.8)	(31.55–32.0)	0.02
Step time (s)	Case1‐intact limb	0.56	0.55	0.01	0.01	(0.56–0.58)	(0.54–0.56)	0.018
	Case1‐amput limb	0.47	0.75	0.01	0.01	(0.46–0.49)	(0.74–0.77)	0.028
	Case2‐intact limb	0.71	0.70	0.00	0.02	(0.70–0.72)	(0.68–0.74)	0.011
	Case2‐amput limb	0.65	0.64	0.00	0.01	(0.64–0.66)	(0.63–0.65)	0.012
Step length (cm)	Case1‐intact limb	22.40	11.27	0.93	0.37	(21.4–23.6)	(10.9–11.6)	0.042
	Case1‐amput limb	19.12	14.20	0.71	0.42	(18.2–19.9)	(13.7–14.6)	0.038
	Case2‐intact limb	8.30	7.67	0.14	0.35	(8.1–8.4)	(7.2–8.0)	0.017
	Case2‐amput limb	8.10	7.65	0.21	0.40	(7.9–8.4)	(7.1–8.0)	0.027
Double stance%	Case1	34.80	33.55	0.98	0.66	(33.9–35.7)	(32.6–34.1)	0.028
	Case2	37.02	35.12	0.97	1.18	(36.0–38.2)	(33.9–36.6)	0.026
Velocity (Km/h)	Case1	1.82	1.60	0.22	0.03	(1.6–2.1)	(1.56–1.65)	0.122
	Case2	0.40	0.30	0.00	0.01	(0.39–0.41)	(0.29–0.32)	0.02
Stride time (s)	Case1	1.36	1.32	0.04	0.03	(1.3–1.4)	(1.29–1.37)	0.033
	Case2	1.36	1.31	0.04	0.02	(1.32–1.42)	(1.29–1.35)	0.031
Cadence (step/min)	Case1	92.18	88.30	2.03	0.75	(89.2–93.5)	(87.4–89.1)	0.022
	Case2	91.97	87.15	2.02	0.82	(89.0–93.4)	(86.2–88.2)	0.022

*Note:* The coefficient of variation (CV).

**Table 4:** Effect size for changes between phases based on d‐index.

		A compared with B			B compared with A1			A compared with A1		
variable	Case and limb	Effect	Upper	Lower	Effect	Upper	Lower	Effect	Upper	Lower
Stance phase %	Case1‐intact limb	7.74	12.21	3.25	0.46	1.85	−0.96	7.10	−2.94	−11.2
	Case1‐amput limb	7.83	−3.30	−12.3	0.65	2.06	−0.80	7.51	11.85	3.14
	Case2‐intact limb	0.16	1.55	−1.22	2.20	−0.31	−4.00	2.53	4.46	0.52
	Case2‐amput limb	1.74	−0.01	−3.39	0.32	1.71	−1.08	1.95	−0.14	−3.65
%Load response	Case1‐intact limb	15.40	−6.86	−24.0	3.42	5.73	1.04	12.73	19.88	5.62
	Case1‐amput limb	0.15	1.23	−1.54	2.40	−0.43	−4.27	2.35	4.20	0.40
	Case2‐intact limb	4.65	−1.70	−7.53	0.54	0.89	−1.94	2.70	−0.62	−4.69
	Case2‐amput limb	2.02	−0.19	−3.75	2.07	−0.22	−3.82	0.21	1.18	−1.59
Mid stance %	Case1‐intact limb	28.85	44.03	13.03	2.34	−0.40	−4.19	24.76	37.81	11.1
	Case1‐amput limb	19.09	−8.56	−29.1	0.35	1.05	−1.74	18.70	−28.6	−8.38
	Case2‐intact limb	2.88	4.94	0.72	0.93	2.38	−0.57	1.83	3.50	0.07
	Case2‐amput limb	0.29	1.10	−1.68	0.59	1.99	−0.85	1.08	0.45	−2.56
Pre swing %	Case1‐intact limb	15.54	−6.92	−24.2	1.18	−2.67	0.38	17.61	−7.88	−26.9
	Case1‐amput limb	5.71	9.12	2.25	1.67	3.29	−0.04	3.95	6.50	1.33
	Case2‐intact limb	0.80	0.67	−2.23	0.37	1.03	−1.76	0.48	0.94	−1.88
	Case2‐amput limb	2.64	4.60	0.58	2.10	‐0.24	‐3.86	4.70	7.60	1.73
Swing phase %	Case1‐intact limb	9.53	−4.11	−14.9	1.93	3.63	0.13	8.11	12.78	3.43
	Case1‐amput limb	10.61	16.62	4.63	1.18	2.67	−0.38	11.65	−5.11	−18.2
	Case2‐intact limb	0.29	1.11	−1.67	1.13	2.61	−0.42	1.21	0.36	−2.72
	Case2‐amput limb*	0.5	0.5	−0.5	0.25	0.25	−0.25	0.5	0.5	−0.5
Step time (s)	Case1‐intact limb	18.99	29.03	8.51	1.56	0.11	−3.15	19.98	30.54	8.97
	Case1‐amput limb	23.44	35.80	10.55	22.16	33.85	9.97	0.98	2.43	−0.54
	Case2‐intact limb	4.29	7.00	1.51	0.26	1.13	−1.65	2.90	4.97	0.74
	Case2‐amput limb	0.26	1.13	−1.65	0.84	0.64	−2.27	0.13	1.51	−1.26
Step length (cm)	Case1‐intact limb	14.51	−6.45	−22.6	15.55	−6.93	−24.2	2.43	4.31	0.45
	Case1‐amput limb	6.88	−2.84	−10.9	8.34	−3.55	−13.1	2.52	4.44	0.51
	Case2‐intact limb	6.57	10.42	2.68	1.73	3.37	0.00	2.01	−0.19	−3.74
	Case2‐amput limb	9.66	−4.17	−15.1	1.38	0.23	−2.93	4.97	−1.87	−8.01
Double stance%	Case1	5.46	−2.12	−8.74	1.48	0.16	−3.05	4.92	−1.84	−7.93
	Case2	4.32	7.03	1.53	1.75	−0.01	−3.39	5.76	9.19	2.27
Velocity (Km/h)	Case1	3.72	−1.20	−6.15	1.39	0.23	‐2.94	4.76	−1.76	−7.70
	Case2	1.72	0.00	−3.35	9.19	−3.95	−14.4	0.04	1.34	−1.42
Stride time (s)	Case1	0.20	1.58	−1.19	0.83	0.65	−2.26	1.27	2.78	−0.32
	Case2	0.25	1.63	−1.15	1.35	0.26	−2.88	0.25	1.63	−1.15
Cadence (step/min)	Case1	0.18	1.21	−1.56	2.53	−0.51	−4.45	2.71	4.70	0.62
	Case2	0.22	1.17	−1.60	3.11	−0.86	−5.28	3.28	5.52	0.96

* Effect size calculated based on G‐index to reduce the effect of trend.

A quantitative analysis of Participant 1 revealed significantly large effect sizes (d‐index > 2.67) across various gait parameters. Changes induced by the intervention for the intact limb showed substantial effects in the stance phase, swing phase, loading response, mid‐stance, pre‐swing, step length, step time, double stance, and velocity, with effect sizes ranging from *d* = 7.74 to 28.85. Similar analyses of the amputated limb also uncovered noteworthy effect sizes related to the intervention in the stance phase, swing phase, mid‐stance, pre‐swing, step length, and step time (*d* = 5.71–23.44).

Follow‐up assessments highlighted significant improvements for the intact limb in both loading response and step length (*d* = 3.42 and 15.55, respectively), as well as for the amputated limb in step length and step time (*d* = 8.34 and 22.16). Longitudinal comparisons between baseline and follow‐up further reinforced the strong effects observed in the intact limb across the stance phase, swing phase, loading response, mid‐stance, pre‐swing, step time, double stance, velocity, and cadence (*d* = 7.10–24.76). Analogous trends were also noted for the amputated limb regarding the stance, swing, mid‐stance, and pre‐swing phases (*d* = 3.95–18.70).

The quantitative analysis demonstrated clinically significant effect magnitudes at various intervention time points (d‐index > 2.67). In Participant 2, the adaptations observed in the intact limb showed robust effect sizes for loading response, mid‐stance, step time, and step length (*d* = 2.88–6.57). Meanwhile, the amputated limb displayed substantial effects in both step length and the double stance phase, with effect sizes of *d* = 9.66 and 4.32, respectively. Longitudinal follow‐up with Participant 2 also revealed noteworthy changes in cadence and velocity, with effect sizes of *d* = 9.19 and 3.11. Furthermore, the pre‐post intervention comparisons highlighted significant effect sizes for the intact limb regarding loading response and step time (*i* = 2.70 and 2.90), as well as for the amputated limb in terms of step length and pre‐swing phase (*d* = 4.97 and 4.70). Comprehensive effect size distributions can be found in Table 4.

To confirm these findings, a one‐way ANOVA with Tukey post‐hoc correction was conducted, revealing statistically significant differences in gait parameters over time. Pairwise comparisons underscored the phase‐specific effects of the intervention, as detailed in Table 5. All analyses adhered to conservative criteria suitable for locomotor rehabilitation studies.

**Table 5:** ANOVA and Tukey tests results.

		*p* value			
variable	Case and limb	ANOVA	Tukey A1‐A	Tukey B‐A	Tukey B‐A1
Stance phase %	Case1‐intact limb	3.57e‐06***	6.50e‐06	1.16e‐05	7.70e‐01
	Case1‐amput limb	2.24e‐06***	8.10e‐06	3.80e‐06	6.21e‐01
	Case2‐intact limb	2.05e‐02	2.76e‐02	9.58e‐01	4.26e‐02
	Case2‐amput limb	4.11e‐02	4.63e‐02	9.52e‐02	8.89e‐01
%Load response	Case1‐intact limb	6.36e‐09***	1.00e‐07	< 1.00e‐10	3.89e‐03
	Case1‐amput limb	1.53e‐02*	2.16e‐02	9.68e‐01	3.16e‐02
	Case2‐intact limb	1.30e‐03**	4.97e‐03	1.61e‐03	7.04e‐01
	Case2‐amput limb	2.88e‐02	9.49e‐01	3.69e‐02	5.97e‐02
Mid stance %	Case1‐intact limb	1.95e‐11***	< 1.00e‐10	< 1.00e‐10	5.13e‐02
	Case1‐amput limb	7.13e‐10***	< 1.00e‐10	< 1.00e‐10	8.72e‐01
	Case2‐intact limb	9.00e‐03**	5.78e‐02	7.89e‐03	4.31e‐01
	Case2‐amput limb	4.22e‐01	4.05e‐01	9.05e‐01	6.41e‐01
Pre swing %	Case1‐intact limb	5.49e‐09***	< 1.00e‐10	< 1.00e‐10	1.90e‐01
	Case1‐amput limb	5.33e‐05***	5.49e‐04	5.10e‐05	1.30e‐01
	Case2‐intact limb	5.22e‐01	8.17e‐01	4.92e‐01	8.40e‐01
	Case2‐amput limb	3.54e‐04***	2.60e‐04	2.26e‐02	2.26e‐02
Swing phase %	Case1‐intact limb	3.69e‐07***	2.40e‐06	5.00e‐07	8.21e‐02
	Case1‐amput limb	2.92e‐08***	1.00e‐07	1.00e‐07	4.98e‐01
	Case2‐intact limb	2.29e‐01	2.25e‐01	8.91e‐01	4.12e‐01
	Case2‐amput limb	1.38e‐05***	1.60e‐05	9.11e‐05	2.15e‐01
Step time (s)	Case1‐intact limb	2.07e‐10***	< 1.00e‐10	< 1.00e‐10	3.08e‐01
	Case1‐amput limb	1.41e‐10***	3.84e‐01	< 1.00e‐10	< 1.00e‐10
	Case2‐intact limb	1.50e‐03**	2.31e‐03	3.96e‐03	9.21e‐01
	Case2‐amput limb	8.06e‐01	9.74e‐01	9.02e‐01	7.95e‐01
Step length (cm)	Case1‐intact limb	1.56e‐09***	1.66e‐01	< 1.00e‐10	< 1.00e‐10
	Case1‐amput limb	7.76e‐07***	5.86e‐02	6.10e‐06	9.00e‐07
	Case2‐intact limb	2.60e‐04***	2.28e‐02	1.91e‐04	1.47e‐02
	Case2‐amput limb	9.01e‐06***	7.29e‐05	9.80e‐06	1.26e‐01
Double stance%	Case1	3.65e‐05***	3.20e‐04	3.72e‐05	1.57e‐01
	Case2	7.39e‐05***	5.95e‐05	1.62e‐03	4.47e‐02
Velocity (Km/h)	Case1	3.98e‐04***	5.25e‐03	3.38e‐04	1.44e‐01
	Case2	2.56e‐02*	9.97e‐01	3.90e‐02	4.38e‐02
Stride time (s)	Case1	2.84e‐01	2.96e‐01	9.57e‐01	4.22e‐01
	Case2	1.66e‐01	9.49e‐01	2.77e‐01	1.80e‐01
Cadence(step/min)	Case1	1.28e‐02*	2.81e‐02	9.52e‐01	1.77e‐02
	Case2	3.69e‐03**	9.32e‐03	9.26e‐01	5.37e‐03

Longitudinal gait analysis revealed notable adaptations related to the intervention across various parameters (repeated‐measures ANOVA, *p* < 0.05). In Participant 1, several measures, including stance phase, loading response, mid‐stance, pre‐swing, swing phase, step time, step length, double stance, velocity, and cadence, showed significant changes in both limbs (*p* < 0.001). For Tukey's post‐hoc analysis pinpointed the effects of the intervention on stance, mid‐stance, pre‐swing, swing, step time, step length, and velocity in both limbs, with additional effects on loading response observed in the intact limb (*q* < 0.01). After assessing the results, we found that changes at the end of the intervention phase significantly affected both legs loading response, the length of steps taken, and walking speed (*q* < 0.05). It suggests that although the intervention was effective, these improvements may only be partially reversible.

For Participant 2, the intervention's effects (Tukey‐adjusted) were noted in the loading response, mid‐stance, step time, and step length of the intact limb, as well as in pre‐swing, swing, step length, and bilateral double stance/velocity of the amputated limb (*q* < 0.05). After the intervention, follow‐up analysis showed that changes linked to end of intervention affected cadence, velocity, intact limb step length, and amputated limb pre‐swing (*q* < 0.05).

## 4 Discussion

In a systematic review in 2025, the application of VR environment simulation to improve balance and gait characteristics in persons with lower extremity amputation was evaluated based on published clinical trials [30]. This systematic review classified research on the use of virtual reality environments to enhance gait and balance in individuals with lower limb amputations into three categories: non‐immersive, semi‐immersive, and fully immersive environments.

Most video gaming systems are used to improve balance [31, 32, 33, 34, 35, 36, 37, 38]. Although, there is studies that used fully immersive VR system and headsets to reduce the phantom pain [30] there was only three studies used fully immersive virtual environment to improve gait and balance in lower limb amputees that two of them had intervention in sitting position and the other one used a fixed exoskeletal robot; so, the user was in an orthostatic position during intervention [39, 40, 41]. All three studies utilized a virtual synthetic environment designed as a game, training tool, or testing ground for users to interact while seated.

However, the CAREN system, which is a semi‐immersive environment that uses motion analysis cameras for real‐time evaluation of whether the user has the potential to walk in it [42, 43, 44, 45, 46, 47, 48, 49], Russell [50, 51, 52, 53, 54, 55, 56, 57]. Also, walking on a treadmill and interaction with an LCD were used for gait training [58, 59, 60, 61]. All of these systems used a synthetic environment.

In this study, we explored the use of a fully immersive VR headset to display a 3D video while participants walked on a treadmill for the first time. Previous studies have not utilized fully immersive systems during walking tasks. As previously mentioned, one key factor that contributes to effective motor control during training is the realistic experience provided by three‐dimensional video in an immersive environment.

### 4.1 Validation of the VR Video

The first phase of this study focused on validating the developed VR video as a walking training program for individuals with transtibial amputation. We employed verbal questions based on three validated questionnaires to assess four aspects of the VR video: presence, immersion, entertainment, and comfort. The VR video achieved 78.75% of presence and 84.37% of immersion scores. Additionally, it received scores of 78.12% for entertainment and 45% for comfort. Overall, the developed VR video for walking training received above half the total score in all aspects except comfort.

The recorded score for entertainment aligns with previous studies on green nature walking experiences in fully immersive VR display systems [62]. Exposure to green nature can reduce walking speed due to users' attention to natural details, including sounds and light [62].

Our goal was to maintain user attention on the visual and auditory signs of the video to enhance presence and engagement [63].

To mitigate the reduction in walking speed, we captured the VR video at the normal walking speed of a person without amputation during the training program for individuals with unilateral transtibial amputation, encouraging them to walk faster [64].

During video capture, we focused on environmental sounds, including children playing in the park and birds in the lawn area, to enhance the visual and auditory experiences. Given that our users had no haptic interaction with the VR environment, the recorded immersion scores were acceptable [63].

The comfort evaluation, conducted using an electronic wearable device comfort tool, yielded the lowest score in this study. Discomfort in the VR environment can stem from three main areas: content, user experience, and hardware [65].

In this study, we utilized a 4 K VR video that did not include any haptic feedback, and we specifically excluded participants who showed signs of motion sickness. Several factors, including headset weight, pressure on the nose, and headwear style, can contribute to discomfort. These might help explain the lower scores observed in the VR‐based walking training program [66].

We assessed reported discomfort through coded questions focusing on movement safety and concerns about potential harm. Thus, it appears that walking training using a fully immersive VR‐based video may induce a sense of anxiety regarding harm among transtibial amputees. Future studies could explore wearing harnesses with enhanced vibrotactile feedback to improve balance and proprioception while reducing anxiety and feelings of vulnerability.

### 4.2 Evaluation of Gait Parameters

The single‐subject study evaluated the gait parameters in two participants over 6 weeks of a VR‐based walking training program and 4 weeks before and 4 weeks after intervention assessments in an ABA style. We selected participants with recent amputations to minimize the effects of walking accommodation skills and reduce variability in results [67].

After 6 weeks, walking speed improved significantly, with the participants achieving a normal range in the 10‐m walk test time for unilateral transtibial amputees [68]. The participant's ten‐meter walk test time decreased by 30 s, reaching clinical significance [69]. This increase in velocity aligns with previous studies results. In a 2012 study, two unilateral transtibial amputees (Participant A, left leg; Participant B, right leg) participated in a 6‐week intervention, which involved two supervised sessions each week featuring 20 min of balance gaming on the Nintendo Wii Fit and 20 min of body‐weight‐supported gait training, leading to significant enhancements across various domains. Spatial‐temporal gait parameters showed positive changes, including increases in gait velocity (A: 23.1%, B: 22.43%) and cadence (A: 13.8%, B: 9.87%), and Participant B also demonstrated improved oxygen uptake efficiency [32].

Gait phase percentage evaluation using inertial sensors elucidates gait asymmetry in individuals with transtibial amputation [28]. Following 6 weeks of intervention, stance phase duration decreased in the intact limb while increasing in the amputated limb; this shift indicates that participants’ gait patterns approached symmetry compared to non‐amputee individuals [28]. Findings from uncontrolled trials involving twelve unilateral transtibial amputees who underwent walking assessments on level ground, a 7° downhill slope, and a 7° uphill slope using the CAREN system and motion capture revealed significant changes in gait mechanics. Temporal analysis showed increased stride and stance times on the prosthetic side. Additionally, symmetry indices indicated a notable decrease in gait symmetry during slope walking, with the step time symmetry index worsening by 43.22% [48].

Loading response duration increased in both limbs, especially in participant number one. This response duration primarily refers to the time required for limb tissues to adapt biomechanically to mechanical loading following amputation [70]. Extended interventions may yield more improvements in the outcomes of participants identified as number two.

In participant number one, the midstance phase duration decreased in the intact limb. The results showed an increase in the amputated limb. During ambulation with a transtibial prosthesis, knee flexion angles are typically higher than those observed in the intact limb; furthermore, midstance knee flexion angle timing is often asymmetrical among transtibial amputees [71]. This change can be related to advancements in walking training techniques [72]. It also can be part of walking adaptation strategies [73]. A study using the CAREN system revealed a significant finding: there was a 200% increase in the peak knee extensor moment in the prosthetic limb during the early stance phase. In contrast, the intact limb experienced a 23.66% decrease at this moment. This suggests that there is a considerable compensatory loading strategy at play, which places greater stress on the residual limb. This raises concerns about the potential long‐term biomechanical effects on the intact side [48]. However, in our study, midstance changes in participant number two were lower.

In Participant Number One, both pre‐swing and swing phase durations increased in the intact limb while decreasing in the amputated limb. Similarly, Participant Number Two exhibited reductions in these variables for both limbs. Previous research has indicated that swing duration is generally longer in amputated limbs than in intact ones, particularly when compared to healthy controls [28]. Thus, the observed decrease in swing duration for amputated limbs suggests positive training outcomes.

After 6 weeks of intervention, step lengths improved bilaterally and approached equality between limbs, consistent with findings from other studies on transtibial amputation [74].

In the first participant, the velocity of gait increased. In the second participant, velocity exhibited a significant increase, too. This increase in velocity corresponds with results from the 10‐m walk test.

In an uncontrolled clinical trial, twelve unilateral transtibial amputees were assessed during ambulation across various complex terrains—including slopes, medial‐lateral translations, and simulated hilly and rocky conditions—using the CAREN system and motion capture technology. Kinematic evaluation revealed that while many primary gait parameters showed no significant differences, exposure to uneven surfaces elicited specific adaptive strategies. Gait symmetry showed improvement, especially in the medial‐lateral translation condition. This condition improved step length and step time symmetry by 8.93% and 6.35%, respectively. The most pronounced adaptations were in step width, which increased substantially in the prosthetic limb (29.85%) during rolling hills and in both limbs on rocky terrain, suggesting a prioritized strategy of widening the base of support for stability. The findings indicate that individuals with unilateral TTA employ distinct, limb‐specific kinematic adjustments, predominantly increasing step width on the prosthetic side, to navigate challenging and dynamic environments safely [55].

The duration of double limb support increased in participant number one after 6 weeks of intervention, while participant number two experienced a decrease. Transtibial prosthetic users often show longer durations of double limb support when compared to individuals without amputations. However, an increase in this duration doesn't automatically signal an improvement in gait patterns; instead, it may point to underlying balance issues.

In a nonrandomized controlled clinical trial comparing twelve unilateral transtibial amputees (TTA) to twelve able‐bodied (AB) controls during level and perturbed walking (including rolling hills, medial‐lateral translations, and rocky terrain), the TTA group demonstrated distinct compensatory strategies to maintain dynamic stability. While there were no notable differences in temporal and spatial measurements, such as step width, step length, and the time of double limb support, the margin of stability remained consistent across groups, and the TTA group consistently walked at a slower speed in the majority of conditions [75].

The time‐related parameters of gait, specifically cadence and stride time, demonstrated changes that stayed within normal ranges [76], which may be related to the participant's age, the length of their residual limbs, and the type of amputation. These modifications are consistent with the findings from the ten‐meter walk test.

The participants in this study were male adults who had recently experienced a traumatic amputation. Our reported data aimed to exclude compensatory walking patterns commonly observed in transtibial amputees [77]. Enhancing gait through additional training without addressing socket fit and residual limb interactions may lead to compensatory movements. We hope future studies will focus on developing walking skills among individuals with transtibial amputation within virtual environments by incorporating interactions through electromyography (EMG) sensors and haptic devices to improve functionality within these control techniques [78, 79].

## 5 Conclusion

A fully immersive virtual reality video‐based walking training program is well‐accepted by users with transtibial prostheses and demonstrates sufficient potential to positively influence gait parameters while improving walking symmetry among individuals with transtibial amputation.

## Author Contributions

**Reza Vahab Kashani:** writing – review and editing (equal), writing – original draft (equal), methodology (equal), validation (equal), investigation (equal), conceptualization (equal), project administration (equal), visualization (equal), funding acquisition (equal), resources (equal). **Mokhtar Arazpour:** writing – original draft (equal), writing – review and editing (equal), project administration (equal), methodology (equal), validation (equal), conceptualization (equal), investigation (equal), supervision (lead), visualization (equal), funding acquisition (equal), resources (equal). **Akbar Biglarian:** data curation (equal), methodology (equal), writing – review and editing (equal), visualization (equal), software (equal), formal analysis (equal). **Fatemeh Keshavarzi:** writing – original draft (equal), writing – review and editing(equal), data curation (equal), visualization (equal), methodology (equal), software (equal), formal analysis (equal), validation (equal).

## Funding

The authors received no specific funding for this work.

## Ethics Statement

This research protocol was approved by the Ethics Committee of the University of Social Welfare and Rehabilitation Sciences. We received the approval code (IR.USWR.REC.1402.107) On December 21, 2023. All participants provided written informed consent prior to enrollment in the study. This research was conducted ethically in accordance with the World Medical Association Declaration of Helsinki.

## Conflicts of Interest

The authors declare no conflicts of interest.

## Transparency Statement

The lead author Mokhtar Arazpour affirms that this manuscript is an honest, accurate, and transparent account of the study being reported; that no important aspects of the study have been omitted; and that any discrepancies from the study as planned (and, if relevant, registered) have been explained.

## Data Availability

The authors confirm that the data supporting the findings of this study are available within the article and its supporting materials.

## 1

Please answer all question with points from 0 to four. The zero means no points and four means full points to each question.

Entertainment:

1. How much was interesting the VR video experience?
2. How much the VR environment in the video motivated you to continue the walking?
3. How much desire do you have to walk with this VR video again?
4. How much you enjoyed during walking training with this VR video?

Immersion:

1. How real did the virtual world seem to you?
2. How much did your experience in the virtual environment seem consistent with your real‐world experience?
3. How much you had the feeling of surrounding with VR environment?
4. How much you engaged with the VR environment you experienced during walking?

Prescence:

1. How much you were unaware of your real environment during VR video training?
2. How aware were you of the real world surrounding while navigating in the virtual world? (i.e. sounds, room temperature, other people, etc.)?
3. Score the amount of focuses you had on the VR environment from zero to four.
4. How much you feel that you are part of the VR environment?

Comfort:

1. How much you had the feeling or Anxiety about probable risk of Harm?
2. How much you feel safety with harness and headset Attachment?
3. How many points you give to your movement easiness?
4. How much safety you felled during perceived change?

## References

[1] B. Yuan , D. Hu , S. Gu , S. Xiao , and F. Song , “The Global Burden of Traumatic Amputation in 204 Countries and Territories,” Frontiers in Public Health 11 (2023): 1258853. 37927851 10.3389/fpubh.2023.1258853 PMC10622756

[2] L. Abou , A. Fliflet , L. Zhao , Y. Du , and L. Rice , “The Effectiveness of Exercise Interventions to Improve Gait and Balance in Individuals With Lower Limb Amputations: A Systematic Review and Meta‐Analysis,” Clinical Rehabilitation 36 (2022): 857–872. 35254152 10.1177/02692155221086204

[3] R. Chen , B. Corwell , Z. Yaseen , M. Hallett , and L. G. Cohen , “Mechanisms of Cortical Reorganization in Lower‐Limb Amputees,” Journal of Neuroscience 18 (1998): 3443–3450. 9547251 10.1523/JNEUROSCI.18-09-03443.1998 PMC6792672

[4] E. Saruco , A. Saimpont , F. Di Rienzo , et al., “Towards Efficient Motor Imagery Interventions After Lower‐Limb Amputation,” Journal of Neuroengineering and Rehabilitation 21 (2024): 55. 38622634 10.1186/s12984-024-01348-3 PMC11017566

[5] F. Malouin , C. L. Richards , A. Durand , et al., “Effects of Practice, Visual Loss, Limb Amputation, and Disuse on Motor Imagery Vividness,” Neurorehabilitation and Neural Repair 23 (2009): 449–463. 19182047 10.1177/1545968308328733

[6] E. Raffin , P. Giraux , and K. Reilly , “The Moving Phantom: Motor Execution or Motor Imagery? *Cortex* ,” A Journal Devoted to the Study of the Nervous System and Behavior 48 (2011): 746–757. 10.1016/j.cortex.2011.02.003 21397901

[7] V. Zotey , A. Andhale , T. Shegekar , and A. Juganavar , “Adaptive Neuroplasticity in Brain Injury Recovery: Strategies and Insights,” Cureus 15, no. 9 (2023): e45873, 10.7759/cureus.45873. 37885532 PMC10598326

[8] T. Sparling , L. Iyer , P. Pasquina , and E. Petrus , “Cortical Reorganization After Limb Loss: Bridging the Gap Between Basic Science and Clinical Recovery,” Journal of neuroscience 44 (2024): e1051232024. 38171645 10.1523/JNEUROSCI.1051-23.2023 PMC10851691

[9] N. Connelly , E. Welsby , B. Lange , and B. Hordacre , “Virtual Reality Action Observation and Motor Imagery to Enhance Neuroplastic Capacity in the Human Motor Cortex: A Pilot Double‐Blind, Randomized Cross‐Over Trial,” Neuroscience 549 (2024): 92–100. 38705350 10.1016/j.neuroscience.2024.04.011

[10] Y. Katsumata , Y. Inoue , S. Toriumi , H. Ishimoto , H. Hapuarachchi , and M. Kitazaki , “Shared Avatar for Hand Movement Imitation: Subjective and Behavioral Analyses,” IEEE Access 11 (2023): 96710–96717.

[11] R. Huang , “Study on the Psychological Impact of Interactive Video Immersion Experience on Viewers,” Highlights in Art and Design 6 (2024): 18–20.

[12] D. Gao , Y. Su , X. Zhang , H. Li , and H. Luo , “The Application of Virtual Reality Meditation and Mind–Body Exercises Among Older Adults,” Frontiers in Psychology 15 (2024), 10.3389/fpsyg.2024.1303880. PMC10985321 38566950

[13] C. Gutiérrez‐Cruz , P. Ulsen‐Morales , F. J. Ruiz‐Perálvarez , et al., “Effects of Fully Immersive Virtual Reality‐Based Training on Cognitive and Motor Functions in Adults With Intellectual Disabilities,” Journal of Intellectual & Developmental Disability 51: 1–11. 10.3109/13668250.2025.2544105 40826519

[14] A. Fusco , M. Iosa , M. C. Gallotta , S. Paolucci , C. Baldari , and L. Guidetti , “Different Performances in Static and Dynamic Imagery and Real Locomotion. An Exploratory Trial,” Frontiers in Human Neuroscience 8 (2014): 760. 25324758 10.3389/fnhum.2014.00760 PMC4183108

[15] K. Fukui , N. Maeda , M. Komiya , et al., “Walking Practice Combined With Virtual Reality Contributes to Early Acquisition of Symmetry Prosthetic Walking: An Experimental Study Using Simulated Prosthesis,” Symmetry 13 (2021): 2282.

[16] R. H. Horner , E. G. Carr , J. Halle , G. Mcgee , S. Odom , and M. Wolery , “The Use of Single‐Subject Research to Identify Evidence‐Based Practice in Special Education,” Exceptional Children 71 (2005): 165–179.

[17] S. Cavenett , S. White , and J. Gomersall , “The Effectiveness of Total Surface Bearing Compared to Specific Surface Bearing Prosthetic Socket Design on Health Outcomes of Adults With a Trans‐Tibial Amputation: A Systematic Review,” JBI Database of Systematic Reviews & Implementation Reports 12 (2014): 233–318. 10.11124/01938924-201210561-00008 27820279

[18] Y. J. Choo , D. H. Kim , and M. C. Chang , “Amputation Stump Management: A Narrative Review,” World Journal of Clinical Cases 10 (2022): 3981–3988. 35665133 10.12998/wjcc.v10.i13.3981 PMC9131228

[19] H. Ahmad and M. Alam , “Effect of Different Prosthetic Feets on the Kinematic of Transtibial Amputee Gait,” Prosthetics and Orthotics International 47 (2023): 314.

[20] J. Gardiner , A. Z. Bari , D. Howard , and L. Kenney , “Transtibial Amputee Gait Efficiency: Energy Storage and Return Versus Solid Ankle Cushioned Heel Prosthetic Feet,” Journal of Rehabilitation Research and Development 53 (2016): 1133–1138. 28355033 10.1682/JRRD.2015.04.0066

[21] M.‐J. Hsu , D. H. Nielsen , S.‐J. Lin‐Chan , and D. Shurr , “The Effects of Prosthetic Foot Design on Physiologic Measurements, Self‐Selected Walking Velocity, and Physical Activity in People With Transtibial Amputation,” Archives of Physical Medicine and Rehabilitation 87 (2006): 123–129. 16401450 10.1016/j.apmr.2005.07.310

[22] O. Mohamed and H. Appling , “5 ‐ Clinical Assessment of Gait☆☆ The authors extend appreciation to Dana Craig, Heather Worden, and Edmond Ayyappa, Whose Work in Prior Editions Provided the Foundation for This Chapter,” in Orthotics and Prosthetics in Rehabilitation (Fourth Edition, eds. K. K. CHUI , M. M. JORGE , S.‐C. YEN , and M. M. LUSARDI (Elsevier, 2020).

[23] T. Shimura , S. Sato , P. Zalar , and N. Matsuhisa , “Engineering the Comfort‐of‐Wear for Next Generation Wearables,” Advanced Electronic Materials 9, no. 9 (2023): 2200512, 10.1002/aelm.202200512.

[24] B. G. Witmer and M. J. Singer , “Measuring Presence in Virtual Environments: A Presence Questionnaire,” Presence: Teleoperators and Virtual Environments 7 (1998): 225–240.

[25] T. W. Schubert , “The Sense of Presence in Virtual Environments: A Three‐Component Scale Measuring Spatial Presence, Involvement, and Realness,” Zeitschrift für Medienpsychologie 15 (2003): 69–71.

[26] D. M. Peters , S. L. Fritz , and D. E. Krotish , “Assessing the Reliability and Validity of a Shorter Walk Test Compared With the 10‐Meter Walk Test for Measurements of Gait Speed in Healthy, Older Adults,” Journal of Geriatric Physical Therapy 36 (2013): 24–30. 22415358 10.1519/JPT.0b013e318248e20d

[27] K. Berner , J. Cockcroft , L. D. Morris , and Q. Louw , “Concurrent Validity and Within‐Session Reliability of Gait Kinematics Measured Using an Inertial Motion Capture System With Repeated Calibration,” Journal of Bodywork and Movement Therapies 24 (2020): 251–260. 33218520 10.1016/j.jbmt.2020.06.008

[28] H. F. Maqbool , I. Mahmood , A. Ali , et al., “Gait Asymmetrical Evaluation of Lower Limb Amputees Using Wearable Inertial Sensors,” Heliyon 10 (2024): e32207. 38975224 10.1016/j.heliyon.2024.e32207 PMC11225666

[29] C. Auerbach and W. Zeitlin . *SSD for R: An R Package for Analyzing Single‐Subject Data* (Oxford Universit Press, 2021), 10.1093/oso/9780197582756.001.0001.

[30] M. Arazpour , F. Keshavarzi , and S. A. Gard , “The Effects of Virtual Reality Environment Simulations on Balance and Gait Rehabilitation in Persons With Lower Extremity Amputation,” Prosthetics and Orthotics International 50, no. 1 (2025): 15–38, 10.1097/pxr.0000000000000428. 40043037

[31] R. L. Abbas , D. Cooreman , H. Al Sultan , M. El Nayal , I. M. Saab , and A. El Khatib , “The Effect of Adding Virtual Reality Training on Traditional Exercise Program on Balance and Gait in Unilateral, Traumatic Lower Limb Amputee,” Games For Health Journal 10 (2021): 50–56. 33533682 10.1089/g4h.2020.0028

[32] B. Imam , W. C. Miller , H. C. Finlayson , et al. “A Telehealth Intervention Using Nintendo Wii Fit Balance Boards and Ipads to Improve Walking in Older Adults With Lower Limb Amputation (Wii. n. Walk): Study Protocol for a Randomized Controlled Trial,” JMIR Research Protocols 3 (2014): e4031. 10.2196/resprot.4031 PMC4376145 25533902

[33] B. Imam , W. C. Miller , L. Mclaren , P. Chapman , and H. Finlayson , “Feasibility of the Nintendo WiiFit™ for Improving Walking in Individuals With a Lower Limb Amputation,” SAGE Open Medicine 1 (2013): 2050312113497942. 26770676 10.1177/2050312113497942 PMC4687776

[34] C. A. Miller , D. M. Hayes , K. Dye , C. Johnson , and J. Meyers , “Using the Nintendo Wii Fit and Body Weight Support to Improve Aerobic Capacity, Balance, Gait Ability, and Fear of Falling: Two Case Reports,” Journal of Geriatric Physical Therapy 35 (2012): 95–104. 22441325 10.1519/JPT.0b013e318224aa38

[35] S. Moorthy , S. Sagar , S. Kumar , et al. “Effect of Virtual Reality Therapy as a Therapeutic Adjunct in Rehabilitation Program Among Traumatic Lower‐Limb Amputees: A Parallel Open‐Label RCT,” Innovative Journal of Medical and Health Science 10 (2020): 797–808.

[36] J. Andrysek , S. Klejman , B. Steinnagel , et al., “Preliminary Evaluation of a Commercially Available Videogame System as an Adjunct Therapeutic Intervention for Improving Balance Among Children and Adolescents With Lower Limb Amputations,” Archives of Physical Medicine and Rehabilitation 93 (2012): 358–366. 22289250 10.1016/j.apmr.2011.08.031

[37] G. Tao , W. C. Miller , J. J. Eng , et al., “Group‐Based Telerehabilitation Intervention Using Wii Fit to Improve Walking in Older Adults With Lower Limb Amputation (WiiNWalk): A Randomized Control Trial,” Clinical Rehabilitation 36 (2022): 331–341. 34841917 10.1177/02692155211061222

[38] G. Tao , W. C. Miller , B. Imam , H. Lindstrom , and M. Payne , “2020. Wii Fit Telerehabilitation for Walking in Older Adults With Lower Limb Amputation (WiiNWalk): An RCT (vol 100, p. E211,” Archives of Physical Medicine and Rehabilitation 101 (2019): 730–731.

[39] V. Z. Pérez , J. C. Yepes , J. F. Vargas , et al. “Virtual Reality Game for Physical and Emotional Rehabilitation of Landmine Victims,” *Sensors journal* 22, no. 15 (2022): 5602. 10.3390/s22155602 PMC9332850 35898105

[40] G. Risso , G. Preatoni , G. Valle , M. Marazzi , N. M. Bracher , and S. Raspopovic , “Multisensory Stimulation Decreases Phantom Limb Distortions and Is Optimally Integrated,” iScience 25 (2022): 104129. 35391829 10.1016/j.isci.2022.104129 PMC8980810

[41] W. Shim , H. Kim , G. Lim , et al. “Implementation of the XR Rehabilitation Simulation System for the Utilization of Rehabilitation With Robotic Prosthetic Leg,” *Applied Sciences* 12, no. 24 (2022): 12659.

[42] E. J. Beltran , J. B. Dingwell , and J. M. Wilken , “Margins of Stability in Young Adults With Traumatic Transtibial Amputation Walking in Destabilizing Environments,” Journal of Biomechanics 47 (2014): 1138–1143. 24444777 10.1016/j.jbiomech.2013.12.011 PMC4050449

[43] R. Beurskens , J. M. Wilken , and J. B. Dingwell , “Dynamic Stability of Individuals With Transtibial Amputation Walking in Destabilizing Environments,” Journal of Biomechanics 47 (2014): 1675–1681. 24679710 10.1016/j.jbiomech.2014.02.033 PMC4043395

[44] B. J. Darter and J. M. Wilken , “Gait Training With Virtual Reality‐Based Real‐Time Feedback: Improving Gait Performance Following Transfemoral Amputation,” Physical Therapy 91 (2011): 1385–1394. 21757579 10.2522/ptj.20100360

[45] J. B. Dingwell , J. P. Cusumano , J. H. Rylander , and J. M. Wilken , “How Persons With Transtibial Amputation Regulate Lateral Stepping While Walking in Laterally Destabilizing Environments,” Gait & Posture 83 (2021): 88–95. 33099136 10.1016/j.gaitpost.2020.09.031 PMC7755758

[46] D. H. Gates , B. J. Darter , J. B. Dingwell , and J. M. Wilken , “Comparison of Walking Overground and in a Computer Assisted Rehabilitation Environment (CAREN) in Individuals With and Without Transtibial Amputation,” Journal of neuroengineering and rehabilitation 9 (2012): 81–90. 23150903 10.1186/1743-0003-9-81 PMC3543217

[47] H. Gholizadeh , E. D. Lemaire , and J. Nantel , “Effects of Unity Prosthetic Elevated Vacuum Suspension System on Minimum Swing Toe Clearance,” Canadian Prosthetics & Orthotics Journal 5 (2022): 36847. 37614477 10.33137/cpoj.v5i1.36847 PMC10443518

[48] H. Gholizadeh , E. D. Lemaire , and E. H. Sinitski , “Transtibial Amputee Gait During Slope Walking With the Unity Suspension System,” Gait & Posture 65 (2018): 205–212. 30558933 10.1016/j.gaitpost.2018.07.059

[49] K. R. Kaufman , M. P. Wyatt , P. H. Sessoms , and M. D. Grabiner , “Task‐Specific Fall Prevention Training Is Effective for Warfighters With Transtibial Amputations,” Clinical Orthopaedics & Related Research 472 (2014): 3076–3084. 24811543 10.1007/s11999-014-3664-0 PMC4160499

[50] E. Russell Esposito , H. S. Choi , B. J. Darter , and J. M. J. P. O. Wilken . “Can Real‐time Visual Feedback During Gait Retraining Reduce Metabolic Demand for Individuals With Transtibial Amputation?,” *Plos One* 12, no. 2 (2017): e0171786. 10.1371/journal.pone.0171786 PMC5300156 28182797

[51] P. H. Sessoms , M. Wyatt , M. Grabiner , et al., “Method for Evoking a Trip‐Like Response Using a Treadmill‐Based Perturbation During Locomotion,” Journal of Biomechanics 47 (2014): 277–280. 24268756 10.1016/j.jbiomech.2013.10.035

[52] E. H. Sinitski , E. D. Lemaire , N. Baddour , M. Besemann , N. Dudek , and J. S. Hebert , “Maintaining Stable Transtibial Amputee Gait on Level and Simulated Uneven Conditions in a Virtual Environment,” Disability and rehabilitation. Assistive technology 16 (2021): 40–48. 31349766 10.1080/17483107.2019.1629186

[53] E. H. Sinitski , E. D. Lemaire , N. Baddour , M. Besemann , N. L. Dudek , and J. S. Hebert , “Fixed and Self‐Paced Treadmill Walking for Able‐Bodied and Transtibial Amputees in a Multi‐Terrain Virtual Environment,” Gait & Posture 41 (2015): 568–573. 25661003 10.1016/j.gaitpost.2014.12.016

[54] J. A. Sturk , E. D. Lemaire , E. H. Sinitski , et al., “Maintaining Stable Transfemoral Amputee Gait on Level, Sloped and Simulated Uneven Conditions in a Virtual Environment,” Disability and Rehabilitation, Assistive Technology 14 (2019): 226–235. 29276850 10.1080/17483107.2017.1420250

[55] G. Thibault , H. Gholizadeh , E. Sinitski , N. Baddour , and E. D. J. P. O. Lemaire . “Effects of the Unity Vacuum Suspension System on Transtibial Gait for Simulated Non‐Level Surfaces,” *Plos One* 13, no. 6 (2018): e0199181. 10.1371/journal.pone.0199181 PMC6002056 29902256

[56] R. C. Sheehan , C. A. Rábago , J. H. Rylander , J. B. Dingwell , and J. M. Wilken , “Use of Perturbation‐Based Gait Training in a Virtual Environment to Address Mediolateral Instability in an Individual With Unilateral Transfemoral Amputation,” Physical Therapy 96 (2016): 1896–1904. 27277497 10.2522/ptj.20150566 PMC5131184

[57] R. C. Sheehan , E. J. Beltran , J. B. Dingwell , and J. M. Wilken , “Mediolateral Angular Momentum Changes in Persons With Amputation During Perturbed Walking,” Gait & Posture 41 (2015): 795–800. 25797789 10.1016/j.gaitpost.2015.02.008 PMC4408235

[58] A. Brandt and H. Huang , “Effects of Extended Stance Time on a Powered Knee Prosthesis and Gait Symmetry on the Lateral Control of Balance During Walking in Individuals With Unilateral Amputation,” Journal of neuroengineering and rehabilitation 16 (2019): 151. 31783759 10.1186/s12984-019-0625-6 PMC6883569

[59] A. Brandt , W. Riddick , J. Stallrich , M. Lewek , and H. H. J. J. O. N. Huang & REHABILITATION, “Effects of Extended Powered Knee Prosthesis Stance Time Via Visual Feedback on Gait Symmetry of Individuals With Unilateral Amputation: A Preliminary Study,” *Journal of Neuroengineering and Rehabilitation* 16 (2019): 1–12. 10.1186/s12984-019-0583-z PMC6737689 31511010

[60] S. Mehdizadeh , H. Nabavi , F. N. Motlagh , and R. J. B. Osqueizadeh , “Effect of Real‐time Video Feedback on the Gait Foot Placement Regularity and Symmetry in Individuals With Trans‐Tibial Amputation: A Case Study,” *preprint* (2018): 381996.

[61] F. M. Alfieri , C. Da Silva Dias , D. M. O. Utiyama , D. V. M. Ayres , and L. R. Battistella , “The Immediate Effect of Exercising in a Virtual Reality Treadmill (C‐Mill) on Skin Temperature of a Man With Lower Limb Amputation,” Case Reports in Vascular Medicine (2023): 7081000, 10.1155/2023/7081000. 39281416 PMC11401695

[62] S. Litleskare , F. Fröhlich , O. E. Flaten , A. Haile , S. Å. Kjøs Johnsen , and G. Calogiuri , “Taking Real Steps in Virtual Nature: A Randomized Blinded Trial,” Virtual Reality 26 (2022): 1777–1793. 35818369 10.1007/s10055-022-00670-2 PMC9261150

[63] S. Jung and R. W. Lindeman , “Perspective: Does Realism Improve Presence in VR? Suggesting a Model and Metric for VR Experience Evaluation,” Frontiers in Virtual Reality 2 (2021): 693327, 10.3389/frvir.2021.693327.

[64] H. R. Batten , S. M. Mcphail , A. M. Mandrusiak , P. N. Varghese , and S. S. Kuys , “Gait Speed as an Indicator of Prosthetic Walking Potential Following Lower Limb Amputation,” Prosthetics & Orthotics International 43 (2019): 196–203. 30112982 10.1177/0309364618792723

[65] L. Thibault , C. Larabi , and D. Meneveaux , “Exploration of Comfort Factors for Virtual Reality Environments,” Electronic Imaging 34 (2022): 393–391.

[66] Y. Yan , K. Chen , Y. Xie , S. Yiming , and Y. Liu . *The Effects of Weight on Comfort of Virtual Reality Devices* (2019): 239–248.

[67] A. B. Wanamaker , R. R. Andridge , and A. M. Chaudhari , “When to Biomechanically Examine a Lower‐Limb Amputee: A Systematic Review of Accommodation Times,” Prosthetics & Orthotics International 41 (2017): 431–445. 28946826 10.1177/0309364616682385

[68] E. H. Beisheim , E. S. Arch , J. R. Horne , and J. M. Sions , “Performance‐Based Outcome Measures Are Associated With Cadence Variability During Community Ambulation Among Individuals With a Transtibial Amputation,” Prosthetics & Orthotics International 44 (2020): 215–224. 32539665 10.1177/0309364620927608 PMC7392798

[69] B. Carse , H. Scott , F. Davie‐Smith , L. Brady , and J. Colvin , “Minimal Clinically Important Difference in Walking Velocity, Gait Profile Score and Two Minute Walk Test for Individuals With Lower Limb Amputation,” Gait & Posture 88 (2021): 221–224. 34119776 10.1016/j.gaitpost.2021.06.001

[70] J. L. Bramley , P. R. Worsley , D. L. Bader , et al., “Changes in Tissue Composition and Load Response After Transtibial Amputation Indicate Biomechanical Adaptation,” Annals of Biomedical Engineering 49 (2021): 3176–3188. 34580782 10.1007/s10439-021-02858-0 PMC8671271

[71] G. Orekhov , A. M. Robinson , S. J. Hazelwood , and S. M. Klisch , “Knee Joint Biomechanics in Transtibial Amputees in Gait, Cycling, and Elliptical Training,” PLoS One 14 (2019): e0226060. 31830082 10.1371/journal.pone.0226060 PMC6907759

[72] E. Pröbsting , M. Bellmann , T. Schmalz , and A. Hahn , “Gait Characteristics of Transtibial Amputees on Level Ground in a Cohort of 53 Amputees—Comparison of Kinetics and Kinematics With Non‐Amputees,” Canadian prosthetics & orthotics journal 2 (2019): 32955. 37614767 10.33137/cpoj.v2i2.32955 PMC10443493

[73] E. C. Prinsen , M. J. Nederhand , and J. S. Rietman , “Adaptation Strategies of the Lower Extremities of Patients With a Transtibial or Transfemoral Amputation During Level Walking: A Systematic Review,” Archives of Physical Medicine and Rehabilitation 92 (2011): 1311–1325. 21714957 10.1016/j.apmr.2011.01.017

[74] Y. Ruiz‐Piragauta , B. P. Torres‐Bello , and E. Camargo‐Casallas , “Analysis of the Spatio‐Temporal Gait Parameters of Transtibial Amputees,” Visión electrónica 1 (2018): 247–253.

[75] B. Imam , W. C. Miller , H. Finlayson , J. J. Eng , and T. Jarus , “A Randomized Controlled Trial to Evaluate the Feasibility of the Wii Fit for Improving Walking in Older Adults With Lower Limb Amputation,” Clinical Rehabilitation 31 (2017): 82–92. 26721873 10.1177/0269215515623601

[76] C. Tudor‐Locke , E. J. Aguiar , H. Han , et al., “Walking Cadence (Steps/Min) and Intensity in 21–40 Year Olds: CADENCE‐Adults,” International Journal of Behavioral Nutrition and Physical Activity 16 (2019): 8. 30654810 10.1186/s12966-019-0769-6 PMC6337834

[77] A. S. Soares , E. Y. Yamaguti , L. Mochizuki , A. C. Amadio , and J. C. Serrão , “Biomechanical Parameters of Gait Among Transtibial Amputees: A Review,” Sao Paulo Medical Journal 127, no. 5 (2009): 302–309. 20169280 10.1590/S1516-31802009000500010 PMC11553117

[78] B. Ahkami , K. Ahmed , A. Thesleff , L. Hargrove , and M. Ortiz‐Catalan , “Electromyography‐Based Control of Lower Limb Prostheses: A Systematic Review,” IEEE Transactions on Medical Robotics and Bionics 5 (2023): 547–562. 37655190 10.1109/tmrb.2023.3282325 PMC10470657

[79] S. Q. Wang , Y. Q. Gao , Z. H. Xu , F. Y. Xu , and L. Yuan , “Effects of Tactile Vibration Feedback System on Balance Function and Walking Ability of a Unilateral Transtibial Amputee With a Prosthesis: A Case Report,” Medicine 99 (2020): e22450. 32991481 10.1097/MD.0000000000022450 PMC7523805

